# Ethanol Positively Modulates Photosynthetic Traits, Antioxidant Defense and Osmoprotectant Levels to Enhance Drought Acclimatization in Soybean

**DOI:** 10.3390/antiox11030516

**Published:** 2022-03-08

**Authors:** Md. Mezanur Rahman, Mohammad Golam Mostofa, Ashim Kumar Das, Touhidur Rahman Anik, Sanjida Sultana Keya, S. M. Ahsan, Md. Arifur Rahman Khan, Minhaz Ahmed, Md. Abiar Rahman, Md. Motaher Hossain, Lam-Son Phan Tran

**Affiliations:** 1 Institute of Genomics for Crop Abiotic Stress Tolerance, Department of Plant and Soil Science, Texas Tech University, Lubbock, TX 79409, USA; mdmerahm@ttu.edu (M.M.R.); skeya@ttu.edu (S.S.K.); 2 Department of Biochemistry and Molecular Biology, Bangabandhu Sheikh Mujibur Rahman Agricultural University, Gazipur 1706, Bangladesh; 3 Department of Agroforestry and Environment, Bangabandhu Sheikh Mujibur Rahman Agricultural University, Gazipur 1706, Bangladesh; ashimbsmrau@gmail.com (A.K.D.); minhaz@bsmrau.edu.bd (M.A.); abiar@bsmrau.edu.bd (M.A.R.); 4 Plant Pathology Division, Bangladesh Rice Research Institute, Gazipur 1701, Bangladesh; anikbge@gmail.com; 5 Department of Agriculture, Bangabandhu Sheikh Mujibur Rahman Science and Technology University, Gopalganj 8100, Bangladesh; smvahsan@bsmrstu.edu.bd; 6 Department of Agronomy, Bangabandhu Sheikh Mujibur Rahman Agricultural University, Gazipur 1706, Bangladesh; arif@bsmrau.edu.bd; 7 Department of Plant Pathology, Bangabandhu Sheikh Mujibur Rahman Agricultural University, Gazipur 1706, Bangladesh; hossainmm@bsmrau.edu.bd

**Keywords:** antioxidant enzymes, gas exchange features, osmotic adjustment, oxidative damage, photosynthesis, reactive oxygen species, water deficiency, water-use-efficiency

## Abstract

Drought is a major environmental threat to agricultural productivity and food security across the world. Therefore, addressing the detrimental effects of drought on vital crops like soybean has a significant impact on sustainable food production. Priming plants with organic compounds is now being considered as a promising technique for alleviating the negative effects of drought on plants. In the current study, we evaluated the protective functions of ethanol in enhancing soybean drought tolerance by examining the phenotype, growth attributes, and several physiological and biochemical mechanisms. Our results showed that foliar application of ethanol (20 mM) to drought-stressed soybean plants increased biomass, leaf area per trifoliate, gas exchange features, water-use-efficiency, photosynthetic pigment contents, and leaf relative water content, all of which contributed to the improved growth performance of soybean under drought circumstances. Drought stress, on the other hand, caused significant accumulation of reactive oxygen species (ROS), such as superoxide and hydrogen peroxide, and malondialdehyde, as well as an increase of electrolyte leakage in the leaves, underpinning the evidence of oxidative stress and membrane damage in soybean plants. By comparison, exogenous ethanol reduced the ROS-induced oxidative burden by boosting the activities of antioxidant enzymes, including peroxidase, catalase, glutathione *S*-transferase, and ascorbate peroxidase, and the content of total flavonoids in soybean leaves exposed to drought stress. Additionally, ethanol supplementation increased the contents of total soluble sugars and free amino acids in the leaves of drought-exposed plants, implying that ethanol likely employed these compounds for osmotic adjustment in soybean under water-shortage conditions. Together, our findings shed light on the ethanol-mediated protective mechanisms by which soybean plants coordinated different morphophysiological and biochemical responses in order to increase their drought tolerance.

## 1. Introduction

Water scarcity is undeniably the most critical environmental constraint limiting agricultural output worldwide. Moreover, growing water demand due to increased population pressure, ongoing climate change-mediated erratic precipitation patterns, and rising temperature will further intensify the drought situation in many regions of the world [1]. Drought stress can trigger a wide array of negative consequences in plants by altering their morphological, physiological, biochemical, cellular, and molecular responses, all of which impede plant growth and development [2,3]. A plethora of studies have reported that water scarcity reduces biomass production and stem elongation, disrupts cellular turgor pressure, restricts water uptake, and interrupts gas exchange performance and nutrient acquisition. Drought stress can also stimulate reactive oxygen species (ROS) accumulation and membrane lipid peroxidation, which ultimately leads to poor growth and even death of plants in severe cases [4,5,6].

Intriguingly, plants have evolved various adaptive strategies to fight against the detrimental consequences of drought [7]. These adaptive strategies include, but are not limited to, increased leaf succulence, enhanced root growth to acquire more water and essential nutrients from the deeper layer of soils, restricted transpirational water loss, retained optimum photosynthetic rate, and improved water-use-efficiency (WUE) [8,9]. In addition, plants can synthesize many compatible compounds, such as proline (Pro), amino acids, and sugars, to maintain osmotic adjustment under drought circumstances [10]. Moreover, plants have evolved complex oxidative stress protection mechanisms to avoid ROS-induced oxidative damage by increasing the synthesis of non-enzymatic antioxidants, such as carotenoids and flavonoids, and stimulating the activities of enzymatic antioxidants, such as glutathione peroxidase (GPX), peroxidase (POD), glutathione *S*-transferase (GST), ascorbate peroxidase (APX), superoxide dismutase (SOD), and catalase (CAT) [11].

Oilseeds have long been regarded as essential components of human diets and the vital raw materials of many industrial applications for the production of pharmaceutical products, oleochemicals, cosmetics, and biofuels [12]. Soybean (*Glycine max*), in particular, is the world’s fourth most important grain crop, accounting for 59% of global oilseed production (www.soystats.com). Soybean acts as a source of 29% edible oil and 70% plant-derived proteins worldwide [6,13]. Importantly, being a legume crop, soybean plays a pivotal role in improving soil fertility through the process known as symbiotic nitrogen-fixation [14,15]. Drought is critical for soybean growth and development and is one of the leading reasons for the soybean yield penalty in arid and semi-arid areas of the world [6,13]. Many strategies, such as gene mining, genetic engineering, and molecular breeding, have been employed to develop soybean varieties with a heightened capacity to survive through water dearth conditions [16]. However, farmers in low-income countries prefer to practice an easy, cost-effective approach that provides immediate agronomic and economic benefits, because biotechnological and breeding research requires more investment and time for developing drought-resilience crops [6,17,18]. Considering these facts, treating plants with cost-effective signaling molecule(s) (SMs) has gained much attention for overcoming drought on numerous agricultural crops, including soybean [6]. Ethanol has emerged as an excellent representative of organic SMs that already showed promising effects in mitigating the adverse impacts of several abiotic stresses, such as chilling stress in rice (*Oryza sativa*) [19] and salt stress in soybean [20], rice, and *Arabidopsis (Arabidopsis thaliana*) [21]. These findings provide a strong rationale for testing the function of ethanol in alleviating the harmful impacts of drought on the economically valuable crop soybean.

In the current research, we intended to investigate whether ethanol could increase the resilience of soybean toward drought stress, as it did in the case of chilling and salt stress tolerance [19,20,21]. With this objective, we examined various morphophysiological and biochemical parameters, including (i) plant growth features and biomass production, (ii) leaf relative water content and succulence as an indicator of water status, (iii) gas exchange parameters, (iv) contents of different photosynthetic pigments, (v) drought-caused ROS generation and membrane lipid peroxidation, (vi) activities and/or levels of different enzymatic and non-enzymatic antioxidants, and (vii) accumulation of several osmoprotectants, to deduce ethanol-mediated drought tolerance mechanisms in soybean.

## 2. Materials and Methods

### 2.1. Plant Materials, Experimental Design, and Treatments

Seed germination and pot-culture of soybean (*Glycine max*) variety (BARI soybean #6) were carried out following the procedures described by Rahman et al. [6]. The average minimum and maximum temperatures during the experimental period were 17 and 34 °C, respectively, with a relative humidity of about 84%. Ten-day-old healthy soybean seedlings grown in pots (eight plants in each pot) were divided into four treatment groups, including (i) water-sprayed well-watered plants (WW), (ii) ethanol-sprayed well-watered plants (Eth), (iii) water-sprayed drought-exposed plants (D), and (iv) ethanol-sprayed drought-exposed plants (Eth + D). Following the methodology of Rahman et al. [6], drought stress was imposed by withholding water irrigation for 8 days, while the control plants were irrigated regularly during the whole experimental period. Plants from ‘Eth’ and ‘Eth + D’ treatment groups were sprayed (8-times in total) with 20-mM ethanol solution (20 mL to each pot), while plants from the ‘WW’ and ‘D’ treatment groups were sprayed (8-times in total) with an equal amount of water every day for a period of 8 days. The applied ethanol dose (20 mM) was selected based on the phenotypes obtained from a small-scale experiment (Supplementary Figure S1). After 8-days of drought exposure, the first trifoliate leaves of soybean plants (19-day-old plants) were harvested to determine numerous parameters associated with soybean morphology, physiology, and cellular biochemistry. The experiment was repeated thrice to ensure the accuracy of the experimental outcome.

### 2.2. Assessment of Growth Parameters

From each treatment, three randomly selected soybean plants were taken to evaluate the growth performance by measuring shoot height, shoot dry weight (DW), root DW, and total DW following the procedures described by Rahman et al. [22]. 

### 2.3. Estimation of Leaf Area, Succulence, Electrolyte Leakage, and Relative Water Content

Total leaf area per trifoliate was estimated according to the following formula reported by Carleton and Foote [23]:

Leaf area (cm^2^) = maximum length × maximum width × 0.75 (correction factor).

Leaf succulence was measured following the comprehensive procedure of Rahman et al. [17]. Leaf electrolyte leakage (EL) percentage was quantified following the protocol of Yang et al. [24] with slight modification. Briefly, 0.2 g of first trifoliate leaves were collected in a 50-mL Falcon tube containing 20 mL of tap water. Initial electrical conductivity (EC_1_) was taken after incubating the samples at 32 °C for 2 h. The samples were heated at 100 °C for 30 min followed by cooling down at room temperature to record final EC (EC_2_). EC of tap water was also measured and referred to as EC_0_. Finally, the EL (%) was calculated using the following equation:

EL (%) = (EC_1_ − EC_0_)/(EC_2_ − EC_0_) × 100.

Leaf relative water content (RWC) was estimated following the procedure outlined by Das et al. [20].

### 2.4. Assessment of Gas Exchange Parameters

An infrared gas analyzer (LI-6400XT, LI-COR Inc., Lincoln, NE, USA) was utilized to estimate the net photosynthetic rate (*Pn*), the stomatal conductance to H_2_O (*g_s_*), the leaf temperature (LT), and the transpiration rate (*E*) as previously described by Rahman et al. [17]. Assessment of photosynthetic parameters was carried out under full sunlight between 11:00 AM and 12:30 PM. WUE parameters, including intrinsic WUE (WUEint) and instantaneous WUE (WUEins), were estimated using *Pn*, *g_s_*, and *E* following the formulae reported in Rahman et al. [17].

### 2.5. Determination of Photosynthetic Pigment Contents

Freshly collected leaves were used to quantify the contents of different photosynthetic pigments, such as chlorophylls (Chls) (e.g., Chl *a*, Chl *b*, and total Chls) and carotenoids, following the protocol outlined by Arnon [25] and Lichtenthaler and Wellbura [26], respectively.

### 2.6. Quantification of the Content of Total Flavonoids

The method proposed by Das et al. [20] was followed to quantify the levels of total flavonoids in the leaf tissues of soybean plants.

### 2.7. Histochemical Analyses of ROS and the Estimation of Hydrogen Peroxide and Malondialdehyde Contents

Freshly harvested leaves were stained using the solutions of nitroblue tetrazolium (NBT) and 3, 3’-diaminobenzidine (DAB) to visualize the accumulations of superoxide (O_2_^•−^) and hydrogen peroxide (H_2_O_2_), respectively, following previously described protocol [20]. The contents of H_2_O_2_ and malondialdehyde (MDA) in the leaf tissues were estimated using a spectrophotometer as outlined by Yu et al. [27] and Kim et al. [28], respectively.

### 2.8. Antioxidant Enzyme Extraction and Assessment of Enzyme Activities

Enzyme extracts were prepared from soybean leaf samples, and the activities of antioxidant enzymes, including CAT (EC: 1.11.1.6), GST (EC: 2.5.1.18), APX (EC: 1.11.1.11), and POD (EC: 1.11.1.7), were determined following the protocol described by Rahman et al. [17].

### 2.9. Measurements of the Levels of Water-Soluble Proteins, Proline, Soluble Sugars, Free Amino Acids, and Carbohydrates

The content of water-soluble proteins was quantified in the extracts used for enzyme activity determination following the method of Bradford [29] using bovine serum albumin as a protein standard. The level of proline (Pro) was determined spectrophotometrically by an acid ninhydrin-based technique, following the procedure reported by Bates et al. [30]. The method used by Somogyi [31] was followed for the quantification of total soluble sugars. The total free amino acid content of the leaf samples was determined with the aid of ninhydrin, in accordance with the protocol proposed by Lee and Takahashi [32]. Following the methodology of Dubios et al. [33], the phenol-sulfuric acid method was used for the determination of total carbohydrate contents in soybean leaves.

### 2.10. Statistical Analysis

Data obtained from four biological replicates per treatment were analyzed by one-way analysis of variance (ANOVA). The means were calculated from biological repeats and compared among the treatments using the least significant difference (LSD) test at *p* < 0.05 with the aid of Statistix 10 software. Different alphabetical letters symbolize significant variations among the control, drought, and ethanol treatments. Heatmap of the fold-change values of different phenotypical and biochemical parameters was created using R studio 1.4.1717.

## 3. Results

### 3.1. Application of 20-mM Ethanol Improved the Phenotypes and Growth Parameters of Soybean Plants Subjected to Drought Stress

**Figure 1:**
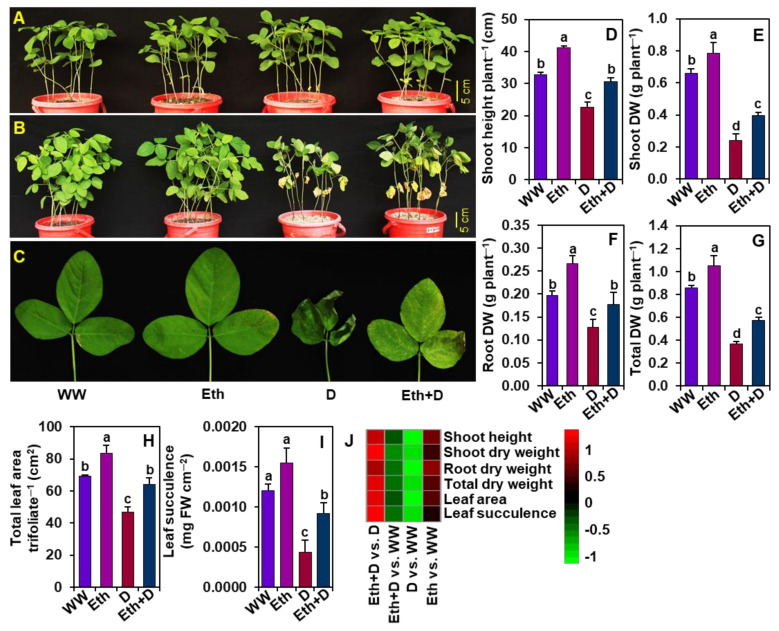
Effect of exogenously supplied 20-mM ethanol on soybean plants exposed to 8 days of water-withholding-induced drought stress. Photographs of soybean plants were taken before (**A**) and after (**B**) exposure to drought stress. (**C**) Close-up photographs of representative soybean leaves (first trifoliate from the bottom of the plant) from each treatment. (**D**) Shoot height, (**E**) shoot DW, (**F**) root DW, (**G**) total DW, (**H**) total leaf area per trifoliate, and (**I**) leaf succulence of soybean plants under different treatments. (**J**) Heatmap of the fold-change values of the aforementioned morphological parameters in soybean plants under different treatments. Bars represent means with standard errors (*n* = 4). Different letters shown above the bars are used to indicate significant differences among the treatments (*p* < 0.05). WW, Eth, D, and Eth + D indicate water-sprayed well-watered plants, ethanol-sprayed well-watered plants, water-sprayed drought-exposed plants, and ethanol-sprayed drought-exposed plants, respectively. DW, dry weight; FW, fresh weight.

To confirm whether ethanol could play a pivotal role in overcoming the drought-mediated pernicious impacts on growth attributes, we recorded plant phenotypes, the height of the shoots, shoot DW, root DW, total DW, trifoliate leaf area, and leaf succulence after 8-days of drought imposition (Figure 1A–J). Phenotypic observations indicated that drought stress caused substantial changes, such as yellowing and semi-drying of leaves in ‘D’ plants, compared with ‘WW’ plants (Figure 1A–C). On the other hand, ‘Eth + D’ plants displayed a noteworthy improvement in phenotypic appearance when compared with that of ‘D’ plants (Figure 1A–C). Shoot height was substantially reduced in ‘D’ plants relative to ‘WW’ plants, whereas external application of ethanol improved shoot height in ‘Eth + D’ plants when contrasted with ‘D’ plants (Figure 1D,J). Likewise, drought stress markedly reduced shoot DW, root DW, and total DW in ‘D’ plants relative to ‘WW’ plants, while ethanol application significantly improved all these DW parameters in ‘Eth + D’ plants in comparison with ‘D’ plants (Figure 1E–G,J). Similarly, relative to ‘WW’ plants, a conspicuous reduction in leaf area and leaf succulence was observed in ‘D’ plants (Figure 1H–J). On the other hand, ethanol application substantially improved leaf area and leaf succulence in ‘Eth + D’ plants when contrasted with ‘D’ plants (Figure 1H–J).

### 3.2. Ethanol Improved Gas Exchange Parameters of Soybean Plants Subjected to Drought Stress

**Figure 2:**
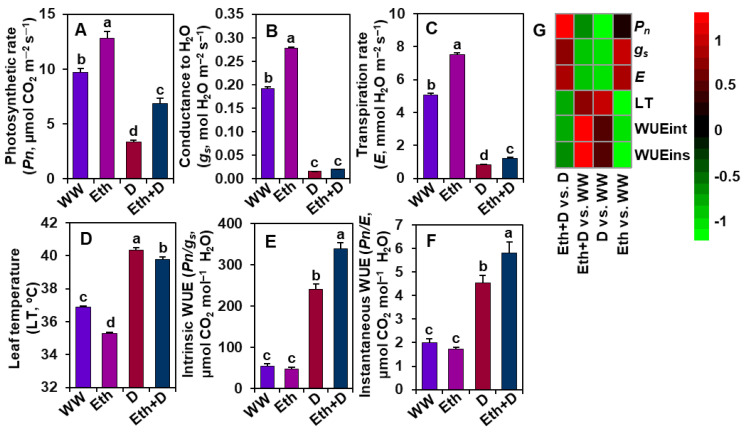
Effect of exogenously supplied 20-mM ethanol on (**A**) photosynthetic rate (*Pn*), (**B**) stomatal conductance to H_2_O (*g_s_*), (**C**) transpiration rate (*E*), (**D**) leaf temperature (LT), (**E**) intrinsic WUE (WUEint), and (**F**) instantaneous WUE (WUEins) of soybean leaves subjected to drought stress for a period of 8 days. (**G**) Heatmap of the fold-change values of the aforementioned parameters in soybean plants under different treatments. Bars represent means with standard errors (*n* = 4). Different letters shown above the bars are used to indicate significant differences among the treatments (*p* < 0.05). WW, Eth, D, and Eth + D indicate water-sprayed well-watered plants, ethanol-sprayed well-watered plants, water-sprayed drought-exposed plants, and ethanol-sprayed drought-exposed plants, respectively. WUE, water-use-efficiency.

*Pn*, *g_s_*, *E*, LT, WUEint, and WUEins were determined to assess whether ethanol has any role in modulating gas exchange parameters under drought stress (Figure 2A–G). We observed that drought stress caused a significant reduction in *Pn*, *g_s_*, and *E*, and an increase in LT, WUEint, and WUEins in ‘D’ plants when compared with those of ‘WW’ plants (Figure 2A–G). By contrast, a noteworthy increase in *Pn*, *E*, WUEint, and WUEins, and a decrease in LT were observed in ‘Eth + D’ plants, relative to ‘D’ plants (Figure 2A,C–G). Nonetheless, ‘Eth’ plants displayed a substantial improvement in *Pn*, *g_s_*, and *E*, and a decrease in LT compared with ‘WW’ plants (Figure 2A–D,G).

### 3.3. Ethanol Improved Photosynthetic Pigment Contents in Soybean Plants Subjected to Drought Stress

**Figure 3:**
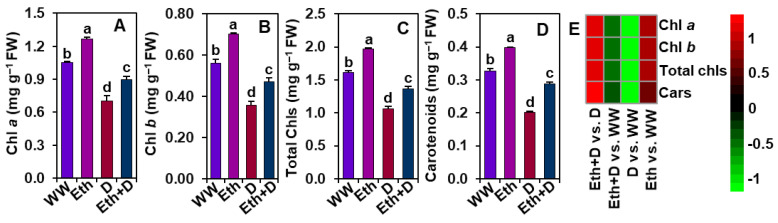
Effect of exogenously supplied 20-mM ethanol on (**A**) Chl *a*, (**B**) Chl *b*, (**C**) total Chls, and (**D**) carotenoid contents in the trifoliate leaves of soybean plants subjected to drought stress for a period of 8 days. (**E**) Heatmap of the fold-change values of the aforementioned parameters in soybean plants under different treatments. Bars represent means with standard errors (*n* = 4). Different letters shown above the bars are used to indicate significant differences among the treatments (*p* < 0.05). WW, Eth, D, and Eth + D indicate water-sprayed well-watered plants, ethanol-sprayed well-watered plants, water-sprayed drought-exposed plants, and ethanol-sprayed drought-exposed plants, respectively. Chl, chlorophyll; Cars, carotenoids; FW, fresh weight.

The contents of different photosynthetic pigments (e.g., Chl *a*, *b*, total Chls, and carotenoids) were determined to examine whether ethanol improves these parameters under drought circumstances (Figure 3A–E). We found that drought stress caused a significant reduction of Chl *a*, *b*, total Chls, and carotenoid levels in ‘D’ plants in comparison with ‘WW’ plants (Figure 3A–E). Contrarily, a substantial improvement in the amounts of Chl *a*, *b*, total Chls, and carotenoids were observed in ‘Eth + D’ plants when equated to those in ‘D’ plants (Figure 3A–E). In comparison with ‘WW’ plants, exogenous ethanol also significantly augmented the levels of total Chls, Chl *a*, Chl *b*, and carotenoids in ‘Eth’ plants (Figure 3A–E).

### 3.4. Ethanol Protected Soybean Plants from Drought-Induced Oxidative Stress

**Figure 4:**
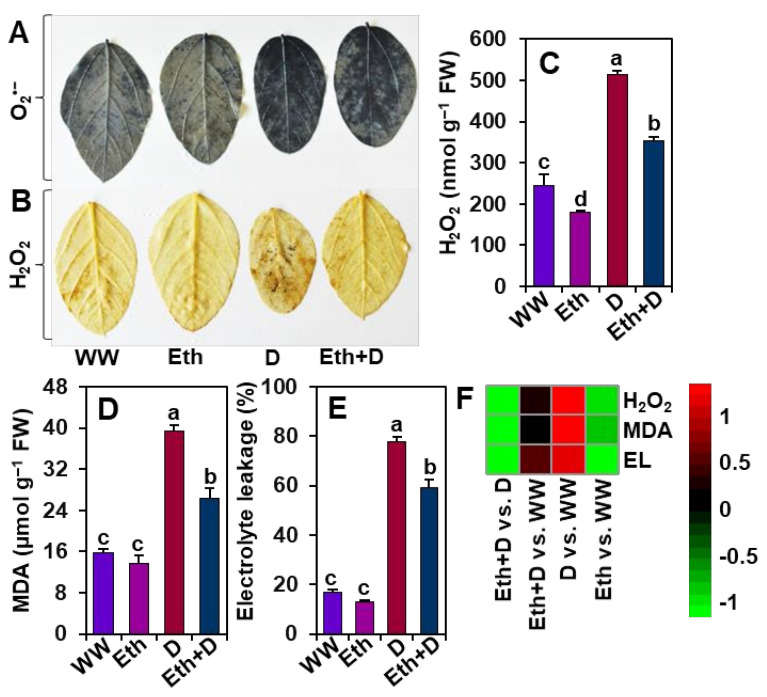
Effect of exogenously supplied 20-mM ethanol on ROS accumulation, and the contents of hydrogen peroxide (H_2_O_2_), malondialdehyde (MDA), and electrolyte leakage in the leaves of soybean plants subjected to drought stress for a period of 8 days. (**A**) Nitroblue tetrazolium (NBT)-staining for detection of superoxide (O_2_^•−^) and (**B**) diaminobenzidine (DAB)-staining for detection of H_2_O_2_. Estimated levels of (**C**) H_2_O_2_, (**D**) MDA, and (**E**) electrolyte leakage in the leaves of soybean plants. (**F**) Heatmap of the fold-change values of the aforementioned parameters in soybean plants under different treatments. Bars represent means with standard errors (*n* = 4). Different letters shown above the bars are used to indicate significant differences among the treatments (*p* < 0.05). WW, Eth, D, and Eth + D indicate water-sprayed well-watered plants, ethanol-sprayed well-watered plants, water-sprayed drought-exposed plants, and ethanol-sprayed drought-exposed plants, respectively. EL, electrolyte leakage; FW, fresh weight; ROS, reactive oxygen species.

To explore the role of ethanol in alleviating drought stress-mediated oxidative stress, we examined ROS generation in soybean leaves by executing histochemical staining of O_2_^•−^ and H_2_O_2_, as well as quantifying the levels of H_2_O_2_, MDA, and EL (Figure 4A–F). In comparison with ‘WW’ plants, drought stress led to a significant accumulation of O_2_^•−^ and H_2_O_2_ in ‘D’ plants, as evidenced by scattered but conspicuous dark-blue spots (O_2_^•−^) and deep dark-brown polymerization patches (H_2_O_2_) (Figure 4A,B). In line with these results, ‘D’ plants also displayed significantly higher levels of H_2_O_2_, MDA, and EL percentage than ‘WW’ plants (Figure 4C–F). By comparison, ‘Eth + D’ plants exhibited substantially lower accumulation of ROS, as well as lower levels of H_2_O_2_, MDA, and EL percentage than ‘D’ plants (Figure 4A–F). ‘Eth’ plants also displayed reduced accumulation of ROS compared with ‘WW’ plants (Figure 4A–C,F). Nonetheless, comparable MDA levels and EL percentages were observed between ‘Eth’ and ‘WW’ plants (Figure 4D–F).

### 3.5. Ethanol Improved Antioxidant Defense in Soybean Plants Subjected to Drought Stress

**Figure 5:**
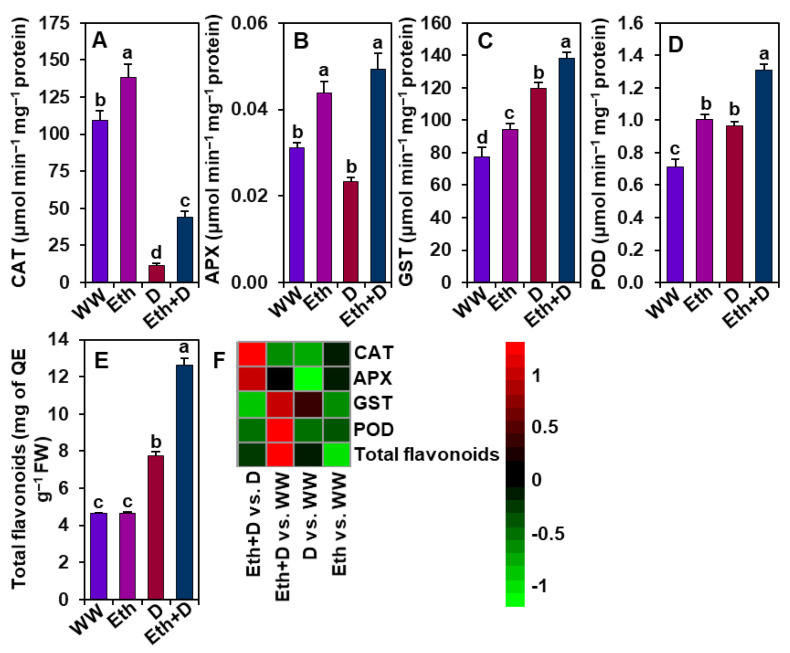
Effect of exogenously supplied 20-mM ethanol on antioxidant defense responses in the leaves of soybean plants subjected to drought stress for a period of 8 days. Activities of (**A**) CAT (catalase), (**B**) APX (ascorbate peroxidase), (**C**) GST (glutathione *S*-transferase), and (**D**) POD (peroxidase) and the content of (**E**) total flavonoids in soybean leaves under different treatment conditions. (**F**) Heatmap of the fold-change values of the aforementioned parameters in soybean plants under different treatments. Bars represent means with standard errors (*n* = 4). Different letters shown above the bars are used to indicate significant differences among the treatments (*p* < 0.05). WW, Eth, D, and Eth + D indicate water-sprayed well-watered plants, ethanol-sprayed well-watered plants, water-sprayed drought-exposed plants, and ethanol-sprayed drought-exposed plants, respectively. FW, fresh weight; QE, quercetin equivalent.

Next, we further examined the activities of some important antioxidant enzymes to evaluate the involvement of ethanol in improving antioxidant defense to alleviate oxidative stress (Figure 5A–F). We found that drought significantly reduced the CAT activity while enhancing the activities of GST and POD, and the number of total flavonoids in ‘D’ plants in comparison with ‘WW’ plants (Figure 5A,C–F). However, we did not observe any significant differences in the activity of APX between ‘D’ and ‘WW’ plants (Figure 5B,F). On the other hand, ethanol supplementation substantially increased the activities of CAT, APX, GST, and POD, and the contents of total flavonoids in ‘Eth + D’ plants in relation to ‘D’ plants (Figure 5A–F). Notably, the activities of CAT, APX, GST, and POD were remarkably improved in ‘Eth’ plants relative to ‘WW’ plants; however, the amounts of flavonoids did not significantly differ between ‘Eth’ and ‘WW’ plants (Figure 5A–F).

### 3.6. Ethanol Improved Water Status, Osmoprotectant Levels, and Water-Soluble Protein Contents in Soybean Plants Subjected to Drought Stress

**Figure 6:**
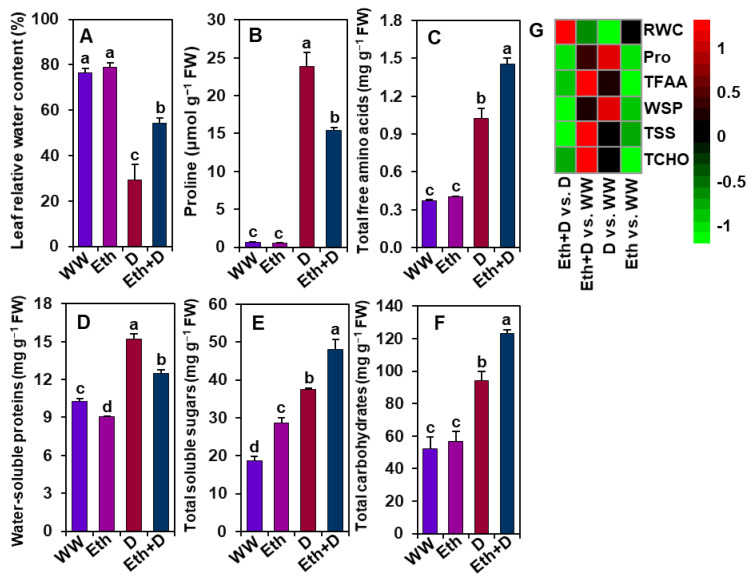
Effect of exogenously supplied 20-mM ethanol on the levels of (**A**) leaf relative water content, (**B**) proline, (**C**) total free amino acids, (**D**) water-soluble proteins, (**E**) total soluble sugars, and (**F**) total carbohydrates of soybean plants subjected to drought stress for a period of 8 days. (**G**) Heatmap of the fold-change values of the aforementioned parameters in soybean plants under different treatments. Bars represent means with standard errors (*n* = 4). Different letters shown above the bars are used to indicate significant differences among the treatments (*p* < 0.05). WW, Eth, D, and Eth + D indicate water-sprayed well-watered plants, ethanol-sprayed well-watered plants, water-sprayed drought-exposed plants, and ethanol-sprayed drought-exposed plants, respectively. FW, fresh weight; Pro, proline; RWC, relative water content; TFAA, total free amino acids; TSS, total soluble sugars; TCHO, total carbohydrates; WSP, water-soluble-proteins.

To confirm whether ethanol assists in restraining water loss under drought stress, the levels of relative water content (RWC), osmoprotectants, water-soluble proteins, and total carbohydrates were determined in soybean plant leaves (Figure 6A–G). Upon drought exposure, ‘D’ plants had a significantly lower level of leaf RWC than ‘WW’ plants (Figure 6A,G). Interestingly, ‘D’ plants displayed higher levels of Pro than ‘WW’ plants (Figure 6B,G). By comparison, ethanol supplementation substantially improved the RWC without further enhancement of Pro in ‘Eth + D’ plants when compared with ‘D’ plants (Figure 6A,B,G). ‘D’ plants also had significantly higher levels of water-soluble proteins, total free amino acids, total soluble sugars, and total carbohydrates than ‘WW’ plants (Figure 6C–G). Notably, the levels of total free amino acids, total soluble sugars, and total carbohydrates were further escalated by exogenous application of ethanol in ‘Eth + D’ plants relative to ‘D’ plants (Figure 6C,E–G). ‘Eth’ plants displayed a significantly higher level of total soluble sugars and lower amount of water-soluble proteins than ‘WW’ plants; however, both ‘Eth’ and ‘WW’ plants showed comparable data for RWC, Pro, total free amino acids, and total carbohydrates (Figure 6A–G).

## 4. Discussion

**Figure 7:**
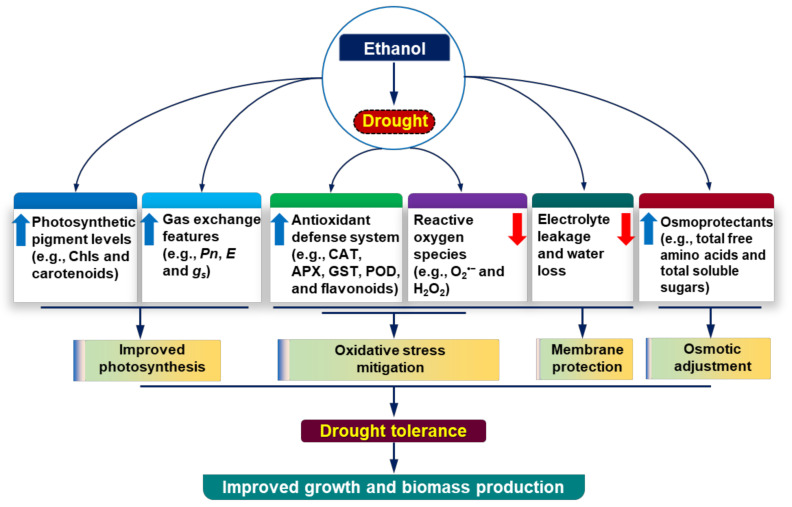
The regulatory roles of ethanol in alleviating drought-induced adverse effects in soybean plants. Foliar application of exogenous ethanol to drought-stressed soybean plants considerably rescued growth phenotypes, partly through the protection of photosynthetic pigments and improvement of gas exchange parameters, which ultimately increased the overall photosynthetic rate to improve growth performance. Exogenous ethanol also triggered the action of the antioxidant defense system by enhancing the activities of enzymatic antioxidants (e.g., CAT, APX, GST, and POD) and the levels of non-enzymatic antioxidants (e.g., total flavonoids), which together contributed to the protection of soybean plants from reactive oxygen species-induced oxidative stress and membrane damage. Additionally, the external application of ethanol enhanced the levels of osmoprotectants (e.g., free amino acids and soluble sugars) to maintain leaf water status for osmotic adjustment under drought circumstances. Upward (blue) and downward (red) arrows indicate increase and decrease, respectively. APX, ascorbate peroxidase; CAT, catalase; Chls, Chlorophylls; *E*, transpiration rate; GST, glutathione *S*-transferase; *g_s_*, stomatal conductance to H_2_O; H_2_O_2_, hydrogen peroxide; O_2_^•−^, superoxide; POD, peroxidase; *Pn*, net photosynthetic rate.

Soybean growth and productivity are severely affected by drought episodes in many parts of the world [5]. Ethanol, an inexpensive chemical (e.g., $1.0 for 7.0 gallons of 20-mM ethanol; $290 for 4 L, Sigma-Aldrich), is known to protect soybean plants from the negative consequences of salinity and chilling stress [19,20,21]. In this study, we also provided evidence that ethanol supplementation enhanced drought tolerance in soybean plants by reducing drought-induced phenotypic aberrations, such as leaf yellowing and senescence, which corresponded with their better growth and biomass production (Figure 1A–G). Under a water-shortage scenario, plants need to forage water and nutrients from deeper layers of soil [34]. Thus, robust root growth can benefit plants by increasing their water absorption spheres under drought conditions. Our study demonstrated that drought stress attenuated total root biomass, whereas ethanol supplementation improved root biomass significantly (Figure 1F). The greater biomass of roots in ethanol-supplemented soybean plants might allow them to absorb more water from the soils [35], thereby contributing to improved soybean growth under water-deficit situations (Figure 7).

When roots perceive a reduction in soil water availability, they convey this environmental constraint as a stress signal toward the shoots [36]. Accordingly, shoots respond to the signal by producing stress hormones like abscisic acid (ABA) to induce stomatal closure for reducing drought-mediated transpirational water loss [37]. It is well known that the complete closure of stomata causes a sharp decline in the photosynthetic rate, which ultimately leads to growth and yield penalty in crops [38]. On the other hand, a partial stomatal closure might help maintain stomatal conductance and transpiration rates in favor of a properly reprogrammed photosynthesis under a water-shortage condition, allowing plants to maximize their WUE [37,39,40,41]. Our results revealed that exogenous ethanol treatment improved photosynthetic rate, stomatal conductance, and transpiration rate, resulting in enhanced WUE (carbon gain to water loss ratio), which might contribute to improving phenotypes and biomass production in drought-stressed soybean plants (Figure 1A–C,E–G and Figure 2A–C,E,F). Furthermore, an improvement in transpiration rate resulted in a decrease in LT, which helped keep the leaves cool, as evidenced by minimal leaf wilting symptoms (Figure 1C and 2D). Together, these results indicated that ethanol played a putative role in modulating gas exchange features to improve soybean drought acclimatization performance under water-limited conditions (Figure 7).

In support of these findings, ethanol-sprayed plants also displayed an improved level of photosynthetic pigments (e.g., Chl *a*, Chl *b*, total Chls, and carotenoids) under both well-watered and water-shortage conditions (Figure 3A–D). These findings suggest that ethanol might be involved in either promoting the synthesis or slowing down the degradation rate of photosynthetic pigments, or both, resulting in an improvement in the net photosynthetic rate and biomass production (Figure 1E–G, Figure 2A and Figure 3A–D). In line with our findings, ethanol-mediated protection of photosynthetic pigments has also been reported in strawberry (*Fragaria ananasa*), soybean, and *Arabidopsis* plants [20,21,42]. It is also worth noting that the greater leaf area per trifoliate in ethanol-supplemented plants might play a positive role in increasing photosynthetic rate (Figure 1H and Figure 2A). Leaf area directly influences plants’ light interception capacity, and consequently, the overall photosynthetic rate and carbon assimilation process [43,44]. Our results highlighted that ethanol spraying significantly increased leaf area compared with water-sprayed plants, supporting the premise of a positive association between increased leaf area and increased photosynthetic rate (Figure 1H and Figure 2A), which coincides with the previous findings of Rahman et al. [6] and Talbi et al. [45].

A number of studies reported that drought-mediated biomass reduction was correlated with the induction of oxidative damage as a result of increased production of ROS, such as O_2_^•−^ and H_2_O_2_ [46,47,48]. In the current study, ‘D’ plants displayed substantial levels of O_2_^•−^, H_2_O_2_, MDA, and EL percentage in the leaves (Figure 4A–E), indicating that drought stress provoked serious oxidative stress and membrane damage in soybean plants. On the other hand, ‘Eth + D’ plants accumulated less O_2_^•−^ and H_2_O_2_, as well as a reduced MDA level and EL percentage (Figure 4A–E), demonstrating that the application of external ethanol mitigated ROS-mediated cell membrane damage in drought-exposed leaves. In support of our results, previous reports also revealed that ethanol was involved in the reduction of salt stress-induced oxidative damage in the leaves of soybean, rice, and *Arabidoposis* [20,21]. In this study, we found a positive correlation of induction of the antioxidant defense with the reduced levels of ROS in ‘Eth + D’ soybean plants (Figure 5). We observed that ‘Eth + D’ soybean leaves maintained an increase in activities of CAT, APX, and POD, which likely contributed to the detoxification of drought-induced H_2_O_2_ [11] (Figure 5A,B,D). Additionally, the greater activity of GST in the leaves of ‘Eth + D’ plants further confirmed the activation of glutathione-dependent H_2_O_2_ removal [49] (Figure 5C). Non-enzymatic antioxidants, such as total flavonoids, were also found to be accumulated in ‘Eth + D’ plants (Figure 5E). Flavonoids are well-recognized for safeguarding cell membrane integrity from oxidative damage by quenching ROS during water-deficit conditions [6,34,50]. These results support that ethanol addition helped soybean plants to maintain a better status of flavonoids, conferring protection against drought-caused oxidative damage. Collectively, ethanol application boosted both enzymatic and non-enzymatic defense to trigger efficient ROS detoxification, thereby diminishing cellular damage for better soybean growth performance under drought stress (Figure 7).

Apart from a vibrant antioxidant defense mechanism, the biosynthesis of low-molecular-weight osmotic compounds, such as Pro, appears to be an important adaptive mechanism for conserving water status under water-shortage situations in plants [10,51]. Our results showed that ’D’ plants accumulated more Pro but retained less RWC in their leaves than control plants (Figure 6A,B). These observations suggest that Pro accumulation in drought-stressed plants was not sufficient to retain water under severe water-deficient environments, which also corroborated with the findings of Dien et al. [52] and Rahman et al. [6]. Alternatively, ‘Eth + D’ plants replenished water loss in the leaves without a substantial increase of Pro contents, implying that Pro accumulation might be an indicator of soybean cellular dehydration (Figure 6A,B), as also observed in other plants under drought stress [53,54]. Interestingly, we also observed rising levels of total free amino acids, total soluble sugars, and total carbohydrates in ‘Eth + D’ plants, in contrast to ‘D’ plants, suggesting that ethanol might compensate water loss independently of Pro but dependently on free amino acids and total soluble sugars (Figure 6A–C,E,F). A number of previous studies have also reported that free amino acids and soluble sugars also played critical roles in maintaining the water status of plants in responses to abiotic stresses, including drought [6,17,22]. Moreover, increased amounts of amino acids and soluble sugars were also known to assure an adequate supply of nitrogen and carbon for sustaining the better metabolism of plants under stressful conditions [55,56,57]. Increased accumulations of total free amino acids and total soluble sugars, as well as their protective roles in counteracting drought-caused adverse effects, have also been reported by Du et al. [58] and Rahman et al. [6] in soybean and Zahoor et al. [59] in cotton (*Gossypium hirsutum*) plants.

## 5. Conclusions

Our study revealed that ethanol, in addition to its growth-promoting effects under normal conditions (Figure 1), improved soybean tolerance to water-deficit stress. We presented the first-ever evidence that supplementation of ethanol improved drought tolerance in soybean by improving root biomass, photosynthetic capacity, and WUE, protecting photosynthetic pigments, reducing ROS-triggered oxidative burst by strengthening antioxidant defense, and uplifting osmoprotectant levels (Figure 7). Nonetheless, it will be interesting to identify the major regulatory pathways that are targeted and modulated by ethanol for developing drought tolerance traits in crop plants. Furthermore, field trials and economic evaluations of ethanol application should also be taken into consideration to verify this cost-effective solution of minimizing drought-induced negative effects and reducing crop yield losses in water-limited adverse conditions.

## Data Availability

Data is contained within the article and Supplementary Materials.

## Supplementary Materials

The following supporting information can be downloaded at: https://www.mdpi.com/article/10.3390/antiox11030516/s1, Figure S1. Effect of different concentrations of ethanol on soybean plants subjected to drought stress for a period of 8 days. D, drought; Eth, ethanol.
