# Supplementary material for: Ethanol Positively Modulates Photosynthetic Traits, Antioxidant Defense and Osmoprotectant Levels to Enhance Drought Acclimatization in Soybean
Source: Antioxidants (Basel). 2022 Mar 8;11(3):516. doi: 10.3390/antiox11030516 (PMC8944470; doi:10.3390/antiox11030516)
Supplement: Supplementary file 1 [file antioxidants-11-00516-s001.zip › antioxidants-1606140-supplementary.pdf]

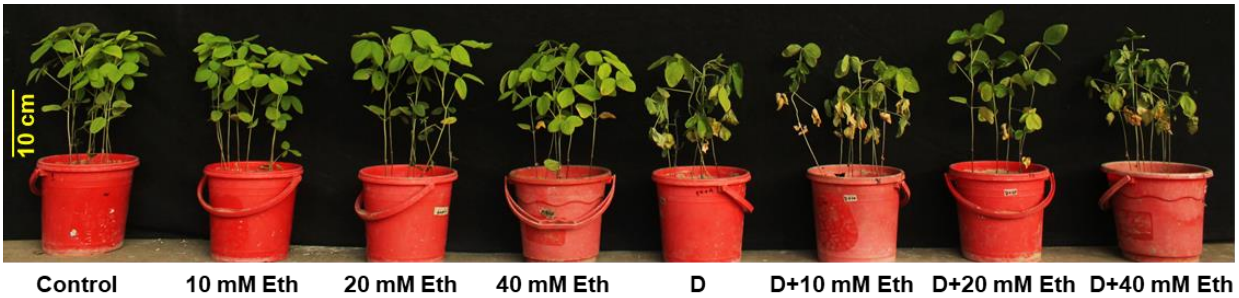

Supplementary Figure S1. Effect of different concentrations of exogenous ethanol on soybean plants subjected to drought stress for a period of 8 days. D, drought; Eth, ethanol.
